# Supplementary figures and images for: Directionality of the injected current targeting the P20/N20 source determines the efficacy of 140 Hz transcranial alternating current stimulation (tACS)-induced aftereffects in the somatosensory cortex
Source: PLoS One. 2022 Mar 24;17(3):e0266107. doi: 10.1371/journal.pone.0266107 (PMC8947130; doi:10.1371/journal.pone.0266107)

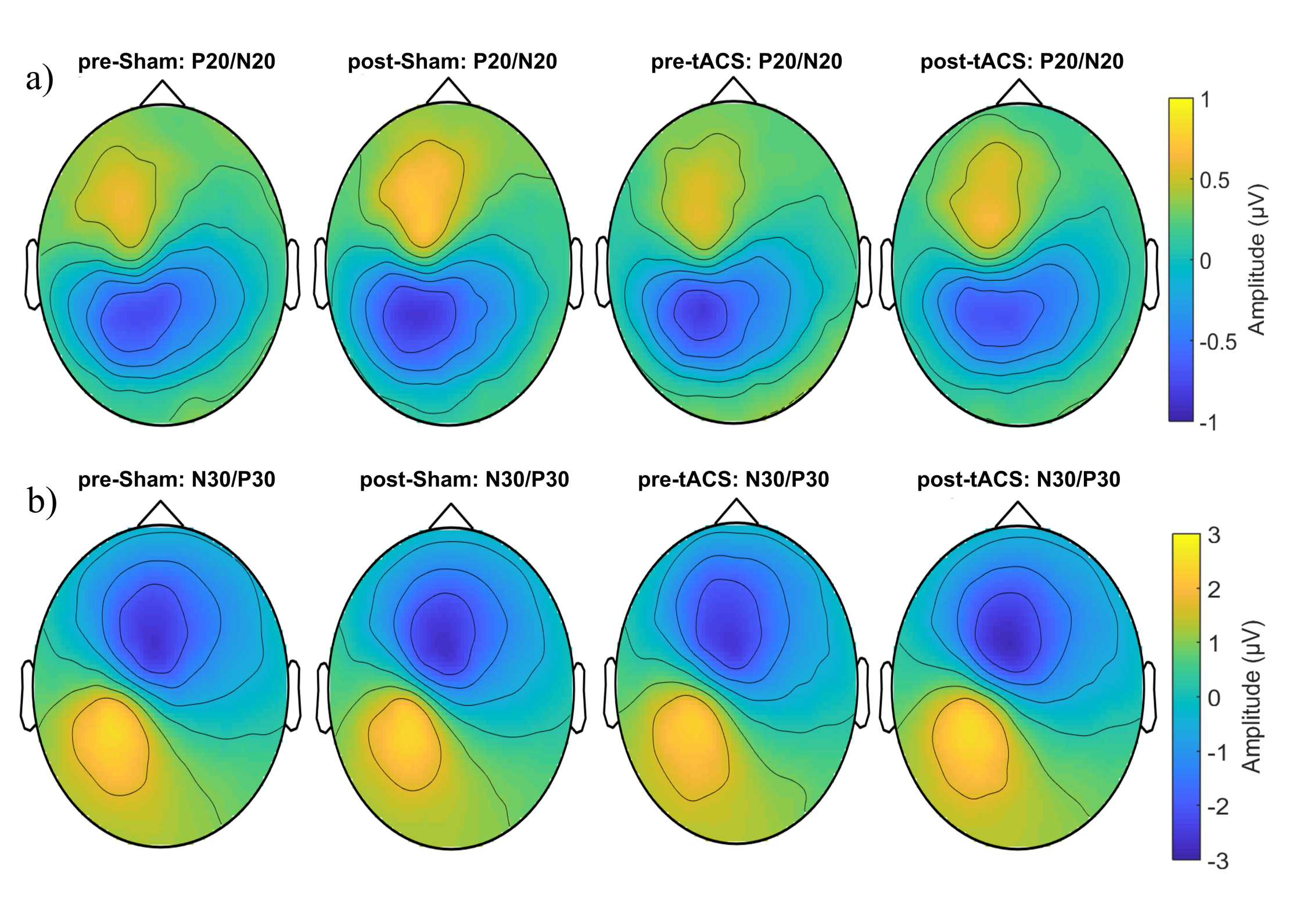

Supplement: S1 Fig — a) The average amplitude of P20/N20. b) The average amplitude of N30/P30. (TIF) [file pone.0266107.s001.tif]
