# Supplementary material for: Directionality of the injected current targeting the P20/N20 source determines the efficacy of 140 Hz transcranial alternating current stimulation (tACS)-induced aftereffects in the somatosensory cortex
Source: PLoS One. 2022 Mar 24;17(3):e0266107. doi: 10.1371/journal.pone.0266107 (PMC8947130; doi:10.1371/journal.pone.0266107)
Supplement: S1 Table — (PDF) [file pone.0266107.s002.pdf]

S1 Table. Number of participants reporting sensations during tACS and sham stimulation

	Yes, n (%)	No, n (%)
1) Sham	11 (37.9)	22 (59.5)
2) tACS	18 (62.1)	15 (40.5)
