# Supplementary material for: Directionality of the injected current targeting the P20/N20 source determines the efficacy of 140 Hz transcranial alternating current stimulation (tACS)-induced aftereffects in the somatosensory cortex
Source: PLoS One. 2022 Mar 24;17(3):e0266107. doi: 10.1371/journal.pone.0266107 (PMC8947130; doi:10.1371/journal.pone.0266107)
Supplement: S2 Table — (PDF) [file pone.0266107.s003.pdf]

S2 Table. Discrimination tasks performance

Frequency difference (Hz)	Correct response (%)			
	Pre-Sham	Post-Sham	Pre-tACS	Post-tACS
1	55.88 ± 17.70	51.76 ± 16.29	58.24 ± 15.10	48.82 ± 23.15
2	60.00 ± 16.58	66.47 ± 14.98	67.06 ± 16.11	65.88 ± 15.44
3	82.94 ± 12.63	75.29 ± 18.75	73.53 ± 18.35	75.29 ± 13.75
4	84.71 ± 17.72	84.12 ± 15.84	82.94 ± 16.11	75.88 ± 15.02
5	79.41 ± 16.00	80.00 ± 17.68	78.82 ± 14.09	76.47 ± 16.18
6	93.53 ± 10.57	89.41 ± 17.84	86.47 ± 19.02	85.29 ± 14.19
7	90.59 ± 20.15	88.24 ± 19.12	95.29 ± 9.43	89.41 ± 12.98
Easy task	87.06 ± 13.56	85.44 ± 14.88	85.88 ±11.72	81.76 ± 11.35
Difficult task	66.27 ±12.30	64.51 ± 14.24	66.27 ± 11.48	63.33 ± 12.42

Values are presented as mean ± SD. tACS = transcranial alternating current stimulation
