# Supplementary material for: Directionality of the injected current targeting the P20/N20 source determines the efficacy of 140 Hz transcranial alternating current stimulation (tACS)-induced aftereffects in the somatosensory cortex
Source: PLoS One. 2022 Mar 24;17(3):e0266107. doi: 10.1371/journal.pone.0266107 (PMC8947130; doi:10.1371/journal.pone.0266107)
Supplement: S3 Table — (PDF) [file pone.0266107.s004.pdf]

S3 Table. Individual stimulation intensity in each stimulation condition.

Participant	Median Nerve Stimulation (mA)		Tactile Stimulation (mA)	
	tACS	Sham	tACS	Sham
P01	16.50	18.00	3.75	3.75
P02	16.50	15.75	2.50	2.50
P03	15.75	13.50	3.75	2.50
P04	18.00	16.50	2.50	2.50
P05	24.00	20.25	2.50	3.75
P06	N/A	N/A	2.50	2.50
P07	12.00	13.50	1.25	2.50
P08	15.75	15.75	2.50	2.50
P09	17.25	17.25	2.50	2.50
P10	14.25	10.50	1.25	2.50
P11	15.75	12.75	2.50	2.50
P12	18.00	9.60	2.50	2.50
P13	23.25	17.25	3.75	3.75
P14	12.75	15.00	2.50	2.50
P15	15.00	10.50	2.50	2.50
P16	14.25	17.25	1.25	1.25
P17	13.50	12.00	3.75	2.50
Mean ± SD	16.41 ± 3.31	14.71 ± 3.08	2.57 ± 0.82	2.65 ± 0.61
t	1.50		-0.30	
p	0.14		0.77	

Abbreviation: tACS = transcranial alternating current stimulation, mA = milliampere, N/A: not available, SD = standard deviation
