# Supplementary material for: Directionality of the injected current targeting the P20/N20 source determines the efficacy of 140 Hz transcranial alternating current stimulation (tACS)-induced aftereffects in the somatosensory cortex
Source: PLoS One. 2022 Mar 24;17(3):e0266107. doi: 10.1371/journal.pone.0266107 (PMC8947130; doi:10.1371/journal.pone.0266107)
Supplement: S4 Table — (PDF) [file pone.0266107.s005.pdf]

S4 Table. Relationship between the angular differences (i.e. differences of the stimulation electrode vector and the source orientation vector) and the SEP changes both at the sensor and source space activities

Condition	r	p
1) Sham		
P20/N20	-0.19	0.48
N30/P30	0.06	0.83
P20/N30 complex	-0.06	0.82
P20/N20 source activity	-0.08	0.76
N30/P30 source activity	-0.05	0.85
2) tACS		
P20/N20	0.13	0.64
N30/P30	-0.32	0.22
P20/N30 complex	-0.21	0.44
P20/N20 source activity	-0.63	0.01*
N30/P30 source activity	-0.57^	0.02*

Abbreviation: tACS = transcranial alternating current stimulation. ^ Spearman correlation; * $p < 0.05$
