# Supplementary material for: Directionality of the injected current targeting the P20/N20 source determines the efficacy of 140 Hz transcranial alternating current stimulation (tACS)-induced aftereffects in the somatosensory cortex
Source: PLoS One. 2022 Mar 24;17(3):e0266107. doi: 10.1371/journal.pone.0266107 (PMC8947130; doi:10.1371/journal.pone.0266107)
Supplement: S5 Table — (PDF) [file pone.0266107.s006.pdf]

S5 Table. Relationship between the angular differences (i.e., differences of the stimulation electrode vector and the source orientation vector) and discrimination task performance

Condition	r	p
1) Sham		
Correct response in easy task (%)	0.25	0.35
Correct response in difficult task (%)	0.23	0.39
Correct response difference in easy task relative to baseline (%)	-.027	0.31
Correct response difference in difficult task relative to baseline (%)	-0.07	0.81
2) tACS		
Correct response in easy task (%)	0.15	0.57
Correct response in difficult task (%)	-0.18	0.52
Correct response difference in easy task relative to baseline (%)	-0.28	0.29
Correct response difference in difficult task relative to baseline (%)	0.15	0.59

Abbreviation: tACS = transcranial alternating current stimulation.
