# Supplementary material for: Directionality of the injected current targeting the P20/N20 source determines the efficacy of 140 Hz transcranial alternating current stimulation (tACS)-induced aftereffects in the somatosensory cortex
Source: PLoS One. 2022 Mar 24;17(3):e0266107. doi: 10.1371/journal.pone.0266107 (PMC8947130; doi:10.1371/journal.pone.0266107)
Supplement: S6 Table — (PDF) [file pone.0266107.s007.pdf]

S6 Table. Relationship between SEP source activities and discrimination task performance

	P20/N20 source activity		N30/P30 source activity	
	r	p	r	p
1) Sham				
Correct response in easy task (%)	-0.12	0.67	0.16	0.57
Correct response in difficult task (%)	-0.21	0.44	-0.07	0.79
Correct response difference in easy task relative to baseline (%)	0.05	0.86	-0.08	0.76
Correct response difference in difficult task relative to baseline (%)	0.34	0.20	0.26	0.34
2) tACS				
Correct response in easy task (%)	-0.32	0.22	-0.13	0.62
Correct response in difficult task (%)	-0.05	0.87	-0.03	0.92
Correct response difference in easy task relative to baseline (%)	0.25	0.36	0.28	0.29
Correct response difference in difficult task relative to baseline (%)	0.13	0.63	-0.18	0.51

Abbreviation: tACS = transcranial alternating current stimulation.
